# Philosophical Transactions of the Royal Society of London. Vol. LXXIII. For the Year 1783. Part I

**Published:** 1784

**Authors:** 


II. *Philoſophical Tranſactions of the Royal Society of London. Vol.* LXXIII. *for the year* 1783. *Part* I. 4to, London, 1783. 246 pages, with 3 copper plates.

1. CONCLUSION *of the experiments and obſervations concerning the attractive powers of the mineral acids. By* Richard Kirwan, *Eſq.* F. R. S.

F. R. S.—Having found, as exactly as he was able, the quantity of each of the mineral acids taken up at the point of saturation by alkalies and earths, and also that taken up by phlogiston, when these acids are converted by it into an aerial form, this ingenious chemist endeavoured to find out how much of these acids was taken up at the point of saturation by each of the metallic substances, and for this purpose procured the most saturated solution possible of each metallic substance soluble in any of the acids. The results of his experiments on this subject are related in the present paper. The advantages resulting from these inquiries are, as the author observes, very considerable, not only in promoting chemical science (which being a physical analysis of bodies, essentially requires an exact determination, as well of the quantity and proportion, as of the quality of the constituent parts of bodies) but also in the practical way. Thus, in the first place, it is well known, that several important processes are very inaccurately described by ancient chymical writers, and even by some of a modern date. They frequently, for instance, describe the acid they employed by reference to the quantity of fixed alkali, earth, or metal, a given quantity of of ſuch acid was capable of neutralizing or diſſolving; now Mr. Kirwan's obſervations immediately inform us of the quantity of real acid capable of performing that effect; the remainder, therefore, muſt have been water; and the quantity of real acid and water being known, the ſpecific gravity is eaſily found by the help of his tables, and then an acid of the ſame ſtrength may be formed. 2dly, The importance of this knowledge in the art of pharmacy is very obvious, eſpecially with regard to medicines formed of metallic ſubſtances, whoſe powers depend on the proportion of their ingredients, and their action on each other. 3dly, This degree of preciſion muſt tend conſiderably to the improvement of the arts of dying and enamelling, the proceſſes by which many of their ingredients are procured being at preſent much too vague. Thus the proceſs at preſent uſed for preparing the precipitate of Caſſius frequently fails, the ſtrength of the acids not being ſufficiently aſcertained. 4thly, The uſes of this knowledge in the examination of mineral waters, and in aſſaying of ores, have been fully proved in Bergman's elaborate treatiſes on that ſubject. But the end which our author thor had principally in view was to aſcertain and meaſure the degrees of affinity or attraction that ſubſiſt betwixt the mineral acids, and the various baſes with which they may be combined, a ſubject of the greateſt importance, as it is upon this foundation that chymiſtry, conſidered as a ſcience, muſt finally reſt. This is what Mr. Kirwan has done with his uſual preciſion, and after a variety of important remarks on chemical attraction he gives us a table of the quantity of baſis taken up by 100 grains of each of the mineral acids, and another table of the affinity of the three mineral acids to metallic ſubſtances. He then treats of the precipitation of metals by each other from the mineral acids; of the abſolute quantity of phlogiſton in metals; of the affinity of metallic calces to phlogiſton; of the affinity of the vitriolic acid and phlogiſton in ſulphur; of ſolutions in the vitriolic, nitrous, and marine acids; of precipitations of and by iron, copper, tin, lead, mercury, biſmuth, nickel, cobalt, antimony, and arſenic.

II. *A deſcription of a ſpecies of Sarcocele of a moſt aſtoniſhing ſize in a black man, in the Iſland of Senegal; with ſome account of its being an endemial*

*mial disease in the county of Galam. By* J. P. Schotte, M. D. *Communicated by Sir* Joseph Banks, *Bart.* P. R. S.—Of this extraordinary disease we formerly gave a short account (Vol. III. p. 426) from memory, after hearing the paper read at the Royal Society; but as the whole of the history is curious and interesting we shall here give it in the words of the author:

" Mr. Bishopp, surgeon in chief of the province of Senegambia (who now resides in London) telling me one day, that he was going to see a poor black man of the Bambara nation, afflicted with a most extraordinary and dreadful disease in his testicles, I accompanied him, being glad of the opportunity of seeing it. We entered the hut, and saw the man lying on a negro bed, elevated about a foot from the ground. He said to Mr. Bishopp, that there was again an ulcer on his scrotum, which had made him take the liberty to request his attendance. I looked at the scrotum, and found it of an astonishing size; but the place where he lay being dark, the hut having no windows, and those people having no candles, he was asked, if he could not walk towards the door, that we might see better.

He anſwered, that he would try; but this was attended with much difficulty. A long cotton ſheet was firſt ſpread on the ground before the bed, which being done, he took, with both his hands, the enormous ſcrotum, moved it gradually on the border of the bed, let it ſlide down gently, and put it into the middle of the ſheet: after this he took the two ends of the ſheet, paſſed them up the fore-part of his body, over his ſhoulders, and had them tied behind his neck. This being done he got up, placing the right-hand upon his right-thigh, and holding the ſheet with the left-hand, and proceeded in this manner, with his knees a little bent, ſlowly towards the door, partly ſliding the ſcrotum on the ground, and partly ſupporting it with his neck by means of the ſheet. I was aſtoniſhed at its enormous ſize, when I ſaw it in the light, and yet I neglected to meaſure it, thinking at the time, as is often the caſe, that I ſhould have opportunities enough to do it; but the ſudden invaſion of the iſland by the French prevented me afterwards from performing it. However, according to my gueſs, and without any exaggeration, the whole maſs might be about two feet and a half long from the os pubis to its lower extremity, and about eighteen inches in diameter acroſs from thigh to thigh.

Its weight I will only ſtate at fifty pounds, as it was eſtimated by Mr. Biſhopp, though I believe it to have been more, and indeed from its dimenſions and from its being a ſolid maſs, it muſt certainly have exceeded that weight. It was of an oblong form, and reſembled in ſome meaſure the ſhape of the ſcrotum of a bull. It felt very hard to the touch, and the ſkin of it was ſo tight, that it could not be pinched by the fingers. The penis was quite hid in the bulk, as generally happens when the ſcrotum is much extended, and may be eaſily comprehended by thoſe who have ſeen large ruptures. The ſkin of the perinæum and of the abdomen was drawn downwards, the navel being nearer to the os pubis than in the natural ſtate. There was a large aperture formed by the ſkin about a foot downwards from the os pubis, rather inclining towards the right-ſide, out of which the urine came, which, however, did not run in a ſtream, but came irregularly from all the interior ſides of the aperture. When he made water, he inclined the maſs, which reſted on the ground, a little forward, and he held a wooden bowl cloſe underneath the aperture, into which the urine was immediately re-

 ceived, ceived, that it might not run along the mafs, and occafion excoriation.

There was an ulcer on the anterior part of the fcrotum, rather towards the left-fide, of about two inches long, and one inch broad and deep. He faid, that it had begun with a puftule or boil, which being broke had gradually increafed to this extent. The pus which came from it was white, thick, and of a good kind. The bottom of it was red, and, when touched with the probe, gave him very acute pain. The edges of it were very callous, and in appearance it did not much differ from an ulcer of a good kind in any other flefhy part of the body. No other remedies were applied to it but thofe generally ufed in common ulcers. It was filled up from the bottom with lint; a pledget of bafilicum was put over it, and the edges were now and then touched with blue vitriol. By thofe means granulations began to fhoot from all fides, the fore filled up gradually, and a cicatrix was formed. He had had fmaller ulcers of this kind in other parts of the fcrotum before this time, which, Mr. Bifhopp told me, he had treated with the fame fuccefs.

The man was rather thin than fat, and might be about fifty years old. He himfelf, like moft blacks, blacks, did not know his age; and if he had pretended to know it, I might, perhaps, not have believed him: for as old age is much reſpected among thoſe people, they are very apt, when they are once paſſed fifty, and have grey hair, to call themſelves older than they really are, in order to command reſpect. His abdomen ſeemed rather empty, and appeared drawn in towards the ſpine; yet I do not think, that any of the inteſtines had deſcended into the ſcrotum, or if any had paſſed down, the annuli of the abdomen muſt have been ſo dilated as not to occaſion the leaſt obſtruction in them; for he never had, to my knowledge, any of thoſe complaints or ſymptoms which attend ruptures. Beſides this, it is to be obſerved, that ruptures are not very common among the blacks about Senegal; indeed I can ſay, that I never ſaw one of them.

Having thus far given an account of what I ſaw myſelf of this remarkable diſeaſe, I ſhall now relate what I have been credibly informed of by other people concerning its beginning and progreſs. The man had been purchaſed up the river as a ſlave, when he was about the age of puberty, and brought down to Senegal, where he was kept as a houſe-ſervant by an opulent inhainhabitant. He was for ſome years healthy and well; but afterwards his teſticles began to ſwell inſenſibly, without inflammation, pain, or any other inconvenience. They increaſed gradually, though ſlowly, and became ſome years after of ſuch a bulk, that he was neither able to walk nor perform his uſual work. That he might, however, not be quite idle, as he was otherwiſe a ſtout and able fellow, he uſed to cut bars of iron into pieces of a foot long, which bear a certain price at Senegal, and go among the blacks like current money. This he could do ſitting with a chiſſel and hammer, and a ſmall anvil placed before him on the ground, his legs bent under him, and the big ſcrotum reſting on the ground. Mr. Biſhopp had ſeen him perform this work for many years; at laſt, however, the ſcrotum increaſed to ſuch a degree, that the great bulk prevented him from doing it any longer. From the time that the diſorder had firſt begun to ſhew itſelf to the time I ſaw him, five-and-twenty years had elapſed; he was alive when I left the iſland in February, 1779, and may be ſo now.

This man was the only one I ever ſaw afflicted with this diſeaſe at Senegal; but I am credibly informed, that it is endemial in a country which goes, goes, among the blacks at Senegal, by the general name of Galam, and of which this man was a native. This country lies eaſt of Senegal, at the diſtance of about nine hundred Engliſh miles, and its inhabitants are called Bambaras. I have been told by thoſe inhabitants of Senegal, who go annually in the rainy ſeaſon in a fleet of ſmall craft to Galam for trade, that this diſeaſe is particularly common among the chiefs or noblemen of that country, who are ſtyled in their own country language Batcherees; and that they have large wooden bowls, fixed on the fore-part of the ſaddle, into which they place the big ſcrotum when they take a ride on horſe-back. Though this latter circumſtance ſeems a little romantic, yet as it has been related to me, not by one but by many, ſeparately and at different times, I give it credit, and I have not the leaſt heſitation to believe, that the diſeaſe is common there. Many of the inhabitants of Senegal have applied to me previouſly to their ſetting off for that country, and aſked me, if I could not give them medicines which would cure that diſorder, with a promiſe, that if they proved ſucceſsful, I might be ſure of a very ample reward of gold; but the improbability of ſucceeding in the reduction of ſuch enormous maſſes to to their priſtine ſtate prevented me from giving them any.

When I was at Fort James in the river Gambia, for a ſhort time in the year 1776, I was told by ſome Marahbuts, or Mahometan prieſts, of the Mandinga nation, that this diſeaſe was now and then to be met with among the chiefs of their nation, and that they knew no cure for it. I have no reaſon to diſcredit their aſſertion, and what makes it more probable is, that the Mandinga and Bambara nations ſeem to be nearly related to one another in outward appearance, cuſtoms, and language, though not entirely in religious matters; for many of the Mandingas are Mahometans, which the Bambaras are not. Their languages reſemble one another ſo nearly that a Bambara from Galam, and a Mandinga from the kingdom of Barrah, which extends from the ſea-coaſt along the north-ſide of part of the river Gambia, can partly underſtand one another. Both nations have alſo a cuſtom of marking their children in various manners by inciſions in the ſkin, and that of filing their fore teeth (inciſores) till they become quite pointed, which I imagine they conſider as being handſome.

As the diſeaſe, according to the information I received, begins with a gradual ſwelling of the teſticles without any pain or inflammation, I am inclined to conſider it as a ſarcocele. Heiſter, in his Surgical Inſtitutions, ſays, that the diſeaſe begins and increaſes moſtly in the ſame manner, when it affects the teſticles themſelves; but that he never ſaw any of them much bigger than a man's fiſt. This difference in the ſize does, in my opinion, not alter the diſeaſe; for we know, that the Bronchocele is hardly known in ſome countries, that it is of a moderate ſize in ſome others, and that in others again it has been ſeen to increaſe to ſuch an enormous bulk as to hang down over the breaſt and belly; yet this difference of ſize does not alter the nature of the diſeaſe, and it ſtill retains the ſame name.

It is difficult to point out the cauſes of ſuch a ſarcocele, as conſiſts in the ſpontaneous tumefaction of the teſticles themſelves; neither do I find any ſatisfactory ones aſſigned by the author I have juſt now quoted; and as I have not been in Galam, I can hardly ſay any thing probable concerning thoſe of the diſeaſe I have deſcribed, I ſhall, however, ſuggeſt the following:

As polygamy is lawful and cuſtomary among the Bambaras as well as among all the other na-

 tions tions about the river Senegal and Gambia, and as the riches and conſequence of a man are eſtimated by the number of wives that he keeps, the chiefs of the people have always a great number of them. I have been told, that the Batcherees of Galam have their victuals moſt immoderately ſeaſoned with Cayenne pepper; and I know myſelf, that the opulent people of the Mandinga nation make the ſame abuſe of it. This may, perhaps, be done with a view to its operating as a provocative; for it has a peculiar effect on the ſeminal veſſels, and will produce erections, attended with a dull pain and turgeſcency in the teſticles: I was therefore inclined to think, that the immoderate uſe of this pepper might partly be the cauſe of this diſeaſe; but then again this could not be the caſe in the man I ſaw at Senegal, where none, or at leaſt very little of it, is uſed.

The moſt probable cauſe of it ſeems to be an hereditary diſpoſition; for, as it only begins to ſhew itſelf about the age of twenty-five or thirty, a man may be father of a great many children before it takes place, and as it ſeems to be confined to families of the principal people of the Bambara nation, it may be, that the man I ſaw afflicted with it at Senegal was deſcended from from ſuch a family, and made a ſlave in his younger years by ſome fatal accident or other, as is often the caſe in thoſe countries."

The other papers in this volume are *a letter from* William Herſchel, Eſq. *to the Preſident*, in which he gives the name of *Georgium Sidus* to the planet he has lately diſcovered.—*On the diameter and magnitude of the Georgium Sidus, by* the ſame.—*A deſcription of a new conſtruction of eye-glaſſes for ſuch teleſcopes as may be applied to mathematical inſtruments, by Mr.* Ramſden.—*An account of ſeveral lunar iris, by* Marmaduke Tunſtall, Eſq. F. R. S. — *An account of an earthquake, by* John Lloyd, Eſq.—*An account of a new Eudiometer, by Mr.* Cavendiſh, F. R. S.—*Experiments upon the reſiſtance of the air, by* R. L. Edgworth, Eſq.—*An anſwer to the objections of* M. de la Lande (concerning the ſolar ſpots) *by* Alex. Wilſon, M. D.—*An account of the earthquakes which happened in Italy, from Feb. to May* 1783, *by Sir* W. Hamilton, K. B. F. R. S.—*Account of the earthquake which happened in Calabria, March* 28, 1783, *by* Count F. Ippolito.—*Account of the black canker caterpillar, which deſtroys the turnips in Norfolk, by* William Marſhall, Eſq.—*Account of wire being ſhortened by light-*

 *ning,*

*ning, by Mr.* E. Nairne, F. R. S.—*An account of ambergriſe, by Dr.* Schwediawer (ſee our 4th vol. p. 97).—*Extract of a regiſter of the barometer, thermometer, and rain, kept at Lyndon, in Rutland,* 1782, *by* Tho. Barker, Eſq.

